# Identification of successive flowering phases highlights a new genetic control of the flowering pattern in strawberry

**DOI:** 10.1093/jxb/erw326

**Published:** 2016-09-24

**Authors:** Justine Perrotte, Yann Guédon, Amèlia Gaston, Béatrice Denoyes

**Affiliations:** ^1^UMR 1332 BFP, INRA, Univ. Bordeaux, F-33140 Villenave d’Ornon, France; ^2^Ciref, Maison Jeannette, 24140 Douville, France; ^3^CIRAD, UMR AGAP and Inria, Virtual Plants, 34095 Montpellier, France

**Keywords:** Flowering phase, *Fragaria* × *ananassa*, genetic control, longitudinal data analysis, multiple change-point model, perpetual flowering.

## Abstract

The genetic control of the switch between seasonal and perpetual flowering has been deciphered in various perennial species. However, little is known about the genetic control of the dynamics of perpetual flowering, which changes abruptly at well-defined time instants during the growing season. Here, we characterize the perpetual flowering pattern and identify new genetic controls of this pattern in the cultivated strawberry. Twenty-one perpetual flowering strawberry genotypes were phenotyped at the macroscopic scale for their course of emergence of inflorescences and stolons during the growing season. A longitudinal analysis based on the segmentation of flowering rate profiles using multiple change-point models was conducted. The flowering pattern of perpetual flowering genotypes takes the form of three or four successive phases: an autumn-initiated flowering phase, a flowering pause, and a single stationary perpetual flowering phase or two perpetual flowering phases, the second one being more intense. The genetic control of flowering was analysed by quantitative trait locus mapping of flowering traits based on these flowering phases. We showed that the occurrence of a fourth phase of intense flowering is controlled by a newly identified locus, different from the locus *FaPFRU*, controlling the switch between seasonal and perpetual flowering behaviour. The role of this locus was validated by the analysis of data obtained previously during six consecutive years.

## Highlight

In perpetual flowering genotypes of strawberry, there are four phases of flowering of which the intensity of the last one is controlled by a newly identified locus.

## Introduction

Flowering is a key step in the plant life cycle, directly linked to the production potential of crop species. The timing and duration of this process are of particular importance for fruit production. In polycarpic perennial plants, flowering usually occurs in a specific seasonal period, the ‘seasonal flowering’ (SF) habit, but some species present genotypes with the ability to initiate flowering during an extended period, consequently offering a lengthened period for flower and fruit production, the ‘perpetual flowering’ (PF) habit, also called continuous flowering (de Camacaro *et al.*, 2002; Albani and Coupland, 2010). Therefore, PF is an attractive agronomical trait, and a better knowledge of its genetic control is a major issue for strawberry breeding.

The genetic control of PF has recently been deciphered and this has highlighted the role of floral repressors in the switch between SF and PF in several species. In *Arabis alpina*, the PF trait is due to a mutation in an orthologue of the *FLOWERING LOCUS C* (*FLC*) floral repressor, *PERPETUAL FLOWERING 1* (*PEP1*) (Wang *et al.*, 2009; Albani *et al.*, 2012), and in the diploid strawberry and rose, to a mutation in an orthologue of the *TERMINAL FLOWER 1* (*TFL1*) floral repressor (Iwata *et al.*, 2012; Koskela *et al.*, 2012). The floral repressor role of *TFL1* was recently confirmed in the cultivated octoploid strawberry (Koskela *et al.*, 2016). In this polyploid strawberry, the PF trait is genetically controlled by the major *FaPFRU* locus, which is non-orthologous to *TFL1* (Gaston *et al.*, 2013). The allelic variant of this *FaPFRU* locus should act as a positive regulator of flowering in the octoploid strawberry genotypes (Perrotte *et al.*, 2016). This locus, which is likely the one described in other studies (Castro *et al.*, 2015; Honjo *et al.*, 2016), includes different flowering genes, and among them an *FT* gene (Perrotte *et al.*, 2016).

Besides the switch between SF and PF, which has been mostly evaluated using retrospective data (e.g. a final count of inflorescences; Gaston *et al.*, 2013), we expect that patterns extracted from longitudinal data (such as durations of flowering) will provide new insights on the dynamics of perpetual flowering. When duration of flowering was considered (e.g. Mathey *et al.*, 2013), data were summarized into binary indicators where genotypes were ranged as SF or PF according to their ability to flower or not in both short and long days. Other studies described periods where flowering is more abundant (Schütz and Milberg, 1997) rather than the dynamics of perpetual flowering.

Until now, the dynamics of developmental traits have not explicitly been considered in genetic studies, whereas recent studies have shown an interest in longitudinal data analyses for deciphering such complex traits (see Dambreville *et al.* (2015) concerning growth and developmental stages in mango growth units and Lièvre *et al.* (2016) concerning developmental phases in *Arabidospsis* rosette). In this setting, the successive measurements of the developmental traits of interest are directly analysed with appropriated statistical models (see Diggle *et al.* (2002) for a general introduction to longitudinal data analysis).

Strawberry stands as an interesting model polycarpic perennial plant for studying the dynamics of flowering and its genetic control. The floral initiation duration is highly variable (Stewart and Folta, 2010) and both SF (also called june-bearing) and PF (also called everbearing) genotypes have been identified among various strawberry species (Hancock *et al.*, 2002). In SF genotypes, the floral initiation is triggered by low temperature and short days in autumn (Battey *et al.*, 1998; Verheul *et al.*, 2007). After a dormancy phase in winter, the autumn-initiated flowers emerge in spring followed by fruiting. In PF genotypes, flowers are initiated continuously throughout the growing season from spring until late autumn and the fruit production period is therefore extended (Savini *et al.*, 2005). Strawberry is also capable of vegetative reproduction by clonal propagation with emergence of elongated branches from basal axillary buds of the crown called primary stolons (the so-called runnering process) (Savini *et al.*, 2008). In PF genotypes, sexual and vegetative reproduction overlap during plant development and are assumed to compete for the same resource pool (e.g. Abrahamson, 1979). Strawberry offers the possibility both to follow the dynamics of the perpetual flowering and to study the relationship between sexual and vegetative reproduction.

Detailed examination of the flowering of perpetual flowering strawberry genotypes over an extended period has indicated that, besides the switch between SF and PF, there are far more complex patterns of flowering (Gaston *et al.* 2013). Therefore, in this study we investigated the dynamics of perpetual flowering and its genetic control based on the number of inflorescences recorded throughout the growing season. The exploratory analysis of our longitudinal flowering data highlighted abrupt changes of flowering intensity through the growing season for PF genotypes. We thus assumed that the flowering pattern of a PF genotype took the form of a succession of well-differentiated stationary flowering phases and analysed this pattern using segmentation models that were in our case multiple change-point models. We designed our study to address the following questions. (i) Can we properly characterize the perpetual flowering pattern by a longitudinal analysis of flowering rate profiles relying on minimum *a priori* assumptions? (ii) Can we identify genetic controls of the dynamics of perpetual flowering using a quantitative trait locus (QTL) approach? (iii) Are these genetic controls related to the previously identified *FaPFRU* locus and stable in other environments?

## Materials and methods

### Plant material

A total of 28 genotypes of the cultivated octoploid strawberry were studied: 21 PF and seven SF genotypes (Supplementary Table S1 at *JXB* online). The 28 genotypes included 26 belonging to a full-sibling F1 population issued from the cross ‘Capitola’ × ‘CF1116’ (Lerceteau-Köhler *et al.*, 2003), and the two parents of this population. Among them, 21 PF genotypes including ‘Capitola’ were chosen for their clear PF phenotype (ranged as PF every year since 2000). Seven SF genotypes including ‘CF1116’ were added as reference for seasonal flowering.

Cold-stored young plants (i.e. harvested in December 2010 and placed in climatic chamber at −1.5 °C until planting) came from the nursery of Ciref (Douville, France, 0° 61′ E and 45° 02′ N, altitude 150 m). They were planted at Ciref on 1 April 2011 (week 14 in 2011). Plants were grown under tunnels on substrate in individual 1L pots, under drip irrigation with fertilized solution. The experiment was performed in a randomized complete block design with five replicate plots for the 28 genotypes (35 plants per genotype).

### Phenotypic data

To evaluate the impact of flowering habit (PF or SF) through the growing season, we studied patterns of emergence of inflorescences and primary stolons in the 28 genotypes. Newly emerged inflorescences were counted weekly from week 16 (14 April) until week 43 (21 October), a total of 28 weeks, and stolons every 2–4 weeks (weeks 19, 21, 23, 26, 29, 32, 35, 39, and 43). Inflorescences and stolons were counted when they visually emerged from the crown. After their emergence, inflorescences were tagged and stolons were cut to ease their counting. To study the relationship between the emergences of inflorescences and stolons, the stolon time indexing was retained. Count data were transformed into growth rates (weekly number of either emerged inflorescences or stolons) since the measurement dates for the stolons were unevenly spaced. In addition, the number of crowns, which are growing branches, was counted at the end of the experiment.

### Statistical models: synchronous segmentation of flowering series for each PF genotype using multiple change-point models

We assumed that the flowering pattern of a PF genotype took the form of a succession of well-differentiated stationary flowering phases where the distribution of the number of weekly emerged inflorescences did not change substantially within each phase, but changed markedly between phases. These flowering patterns have been analysed using segmentation models applied to each PF genotype. We thus assumed that the flowering phases were common for the different plants measured for a given genotype and used multiple change-point models for the synchronous segmentation of the flowering series of the different plants within a genotype. For each PF genotype, the data to be segmented thus consisted of a multivariate series of length 28 (the number of measurement dates) where each variable corresponded to a plant.

Because the number of weekly emerged inflorescences was between 0 and 8 and the frequencies were high except for the three largest values (6, 7 and 8), we chose to consider this variable as categorical with six possible categories, the last one corresponding to the grouping of the values ≥ 5 (the frequency of more than five weekly emerged inflorescences was only 33 compared with a sample size of 19 852—i.e. the cumulative length of the 709 flowering series) for multiple change-point model estimation. We thus directly estimated probability masses for the six possible categories within a given flowering phase. The rather large sample sizes (between 32 and 35 plants—except 22 for the parent ‘Capitola’—to be multiplied by the length of the flowering phase in weeks) justified the direct estimation of probability masses for the six possible categories.

The multivariate flowering series of length *T*, $x=x_{1},\ldots ,x_{T}$ corresponding to a given PF genotype is indexed by the successive weeks of observation (with the convention that the first week is 1 for notational convenience). We assumed that there existed J−1 change points $\tau_{1}<\cdots <\tau_{J-1}$ (with the convention $\tau_{0}=1$ and $\tau_{J}=T+1)$ such that the distribution of the number of weekly emerged inflorescences for the different plants did not change between two successive change points. The J−1 change points $\tau_{1},\ldots ,\tau_{J-1}$ define a unique segmentation $s=s_{1},\ldots ,s_{T}.$ The problem is then to estimate the parameters of this multiple change-point model: the number of flowering phases *J*, the position of the J−1 change points $\tau_{1},\ldots ,\tau_{J-1}$ and the distribution of the number of weekly emerged inflorescences for each flowering phase *j*. Let θ denote the parameters of the distributions attached to the successive flowering phases (i.e. the probability masses for the possible numbers of weekly emerged inflorescences) and LJ(s,x; θ^) the likelihood of the segmentation **s** of the observed multivariate series **x**. The J−1 change points ${\hat{\tau }}_{1},\ldots ,{\hat{\tau }}_{J-1}$, which correspond to the optimal segmentation $s^{*}$ into *J* flowering phases, were estimated using a dynamic programming algorithm (Auger and Lawrence, 1989) that solves the following optimization problem:

$${\hat{\tau }}_{1},\ldots ,{\hat{\tau }}_{J-1}=\arg\underset{s}{\max}\log L_{J}(s,x;\hat{\theta }).$$

We used the integrated completed likelihood (ICL) criterion (Rigaill *et al.*, 2012), a model selection criterion dedicated to the segmentation objective, to determine the number of flowering phases. For each number of flowering phases, the following quantity was computed:

$${\text{ICL}}_{J}=2\log L_{J}(x)-d_{J}\log T-2H(S\text{|}X=x;J),$$ (1)

where LJ(x)=∑sLJ(s,x; θ^) is the likelihood of the all the possible segmentations in *J* flowering phases of the observed multivariate series **x**, $d_{J}$ is the number of free parameters of a *J*-flowering-phase model and $H(S\text{|}X=x;J)=-\sum_{s}P(S=s\text{|}X=x)\log P(S=s\text{|}X=x)$ is the entropy of the segmentation **S** in *J* flowering phases for the observed series **x**. The principle of this penalized likelihood criterion consists in making a trade-off between an adequate fitting of the model to the data (given by the first term in Eqn 1) and a reasonable number of parameters to be estimated (controlled by the second term in Eqn 1). The ICL criterion adds an entropy term in the penalty and is expected to favour models that give rise to the less ambiguous segmentation of the observed series **x** in flowering phases. The log-likelihood term $\log L_{J}(x)$ and the entropy term H(S|X=x;J) involved in the ICL criterion can be efficiently computed using the smoothing algorithm proposed by Guédon (2013, 2015). The posterior probability of the *J*-flowering-phase model $M_{J},$ given by

$$P\left(M_{J}\text{|}x\right)=\frac{\exp\left(\frac{1}{2}{\text{ICL}}_{J}\right)}{\sum_{K=1}^{J_{\max}}\exp\left(\frac{1}{2}{\text{ICL}}_{K}\right)},$$

can be used to assess the relative merits of the models considered.

Once the number of flowering phases *J* had been selected for a given PF genotype, the multivariate series was optimally segmented into *J* flowering phases. The posterior probability of the optimal segmentation $s^{*}$ given by

P(s*|x;J)=LJ(s*,x; θ^)/∑sLJ(s,x; θ^),

can be efficiently computed using the smoothing algorithm proposed by Guédon (2013). The assessment of multiple change-point models thus relies on two posterior probabilities:

(i) posterior probability of the selected *J*-flowering-phase model $M_{J},$ $P(M_{J}\text{|}x),$ deduced from the ICL criterion computed for a collection of multiple change-point models for $J=1,\ldots ,J_{\max},$i.e. weight of the *J*-flowering-phase model among all the possible models between 1 and $J_{\max}$ flowering phases;
(ii) posterior probability of the optimal segmentation $s^{*}$ in *J* flowering phases $P(s^{*}\text{|}x;J),$ i.e. weight of the optimal segmentation among all the possible segmentations in *J* flowering phases.

We used various diagnostic tools, and in particular the dynamic programming algorithm for computing the top *N* most probable segmentations in *J* flowering phases proposed in Guédon (2013), to assess the synchronous segmentation assumption (see an illustration with alternative segmentations in Supplementary Table S1). We also visualized the posterior probabilities of entering flowering phase *j* at time *t*, $P(S_{t}=j,S_{t-1}=j-1\text{|}x;J),$ and the posterior probabilities of being in flowering phase *j* at time *t*, $P(S_{t}=j\text{|}x;J),$ for each time *t* and each flowering phase *j* for *J*-flowering-phase models of interest (see illustrations in Guédon, 2013). These posterior probability profiles computed using the smoothing algorithm (Guédon, 2013) are particularly useful for assessing change-point position uncertainty.

Multiple change-point models and associated statistical methods are implemented in the StructureAnalysis package that is part of the OpenAlea platform (freely available at http://openalea.gforge.inria.fr).

### Genetic maps, QTL detection and genetic effect of one marker linked to the QTL

The initial population, to which the 26 genotypes and the two parents ‘Capitola’ and ‘CF1116’ belonged, comprised 213 individuals. Female and male genetic maps of this population were previously developed using data obtained from the cross ‘Capitola’ × ‘CF1116’ (Rousseau-Gueutin *et al.*, 2008; Lerceteau-Köhler *et al.*, 2012). QTL detection was performed by composite interval mapping (Zeng 1993, 1994) using QTL Cartographer software (Basten *et al.*, 1997) separately for each parent as previously described by Lerceteau-Köhler *et al.* (2012). A total of 1000 permutations of the phenotypic data were performed for each trait to determine a *P*<0.05 logarithm of odds (LOD) signiﬁcance threshold level: from 5.2 to 9.3 for female data and from 5.4 to 9.1 for male data. QTL analyses were performed separately for the number of inflorescences or stolons newly emerged during the considered period.

The genetic effect of the marker EMFv020_146 linked to the QTL (H and A denote, respectively, presence and absence of this marker) was studied on the entire segregating population ‘Capitola’ × ‘CF1116’, for which the flowering observed from September to October or November has been recorded for six consecutive years, from 2004 to 2009.

## Results

### Exploration of the flowering and the runnering patterns of the genotypes

**Fig. 1.**
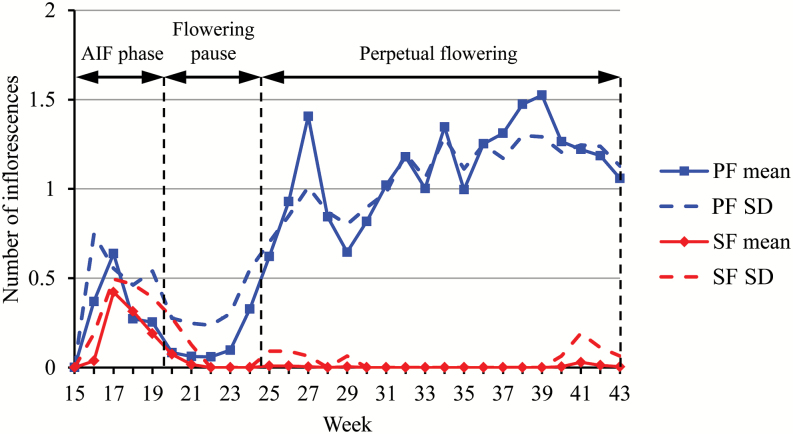
Weekly mean number of emerged inflorescences (and associated standard deviation, SD) for perpetual flowering (PF) and seasonal flowering (SF) individuals. AIF: autumn-initiated flowering.

Perpetual flowering is a key component for wild plants as it makes possible the production of seeds over a long period, and for crop plants as it enlarges the fruit production period. So far, this trait has been evaluated at the end or at a specific time of flowering without considering its dynamics through the growing season. In order to investigate the dynamics of flowering, 21 PF and seven SF genotypes of strawberry were phenotyped weekly from planting until late autumn. After discarding plants that died before the end of the assay, the weekly mean numbers of emerged inflorescences (and associated standard deviations) were computed for the 21 PF genotypes based on the corresponding 709 plants and for the seven SF genotypes based on the corresponding 242 plants (Fig. 1 and Supplementary Fig. S1).

The first phase of emergence of inflorescences, between weeks 16 and 19, was synchronized whatever the flowering habit, PF or SF (Fig. 1). It corresponded to the period of emergence of inflorescences initiated in autumn the previous year and will be referred to as the autumn-initiated flowering (AIF) phase. Then flowering slowed down drastically and almost ceased between weeks 20 and 23. Hereafter, this period will be referred to as the flowering pause. Only PF genotypes still produced new inflorescences after week 23 (or 24) corresponding to the PF phase. In this PF phase, the numbers of weekly emerged inflorescences were rather similar between the genotypes until week 30 and then diverged markedly between genotypes.

**Fig. 2.**
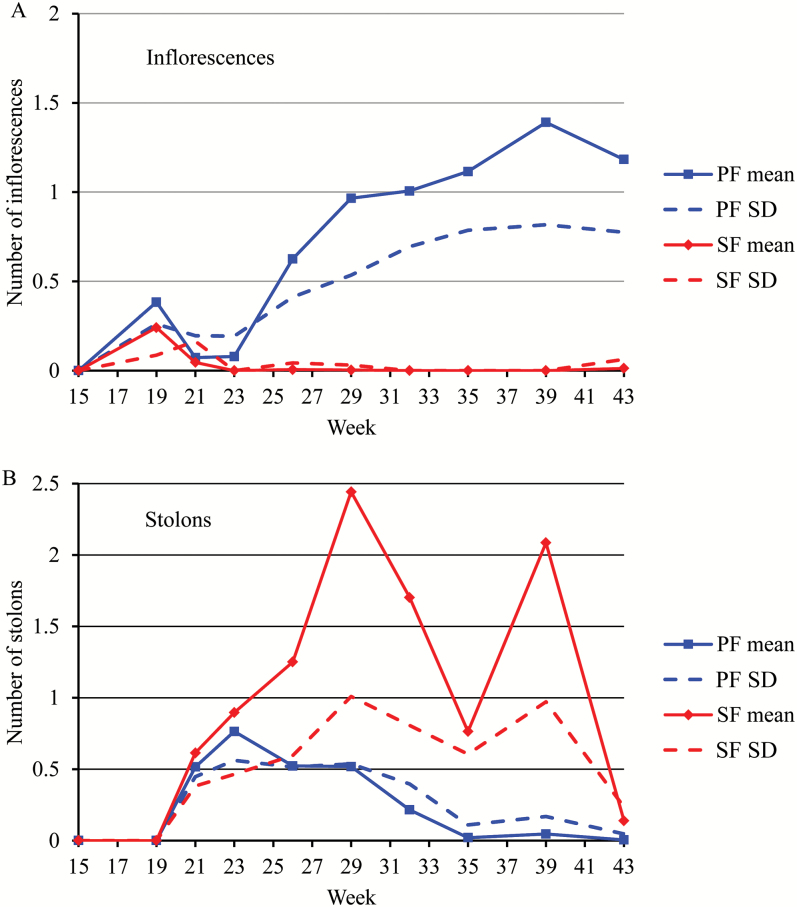
Pointwise mean number of weekly emerged inflorescences (A) and mean number of weekly emerged stolons (B) (and associated standard deviation, SD) for perpetual flowering (PF) and seasonal flowering (SF) individuals. The common indexing of these mean and standard deviation series is the sparser stolon time indexing.

**Fig. 3.**
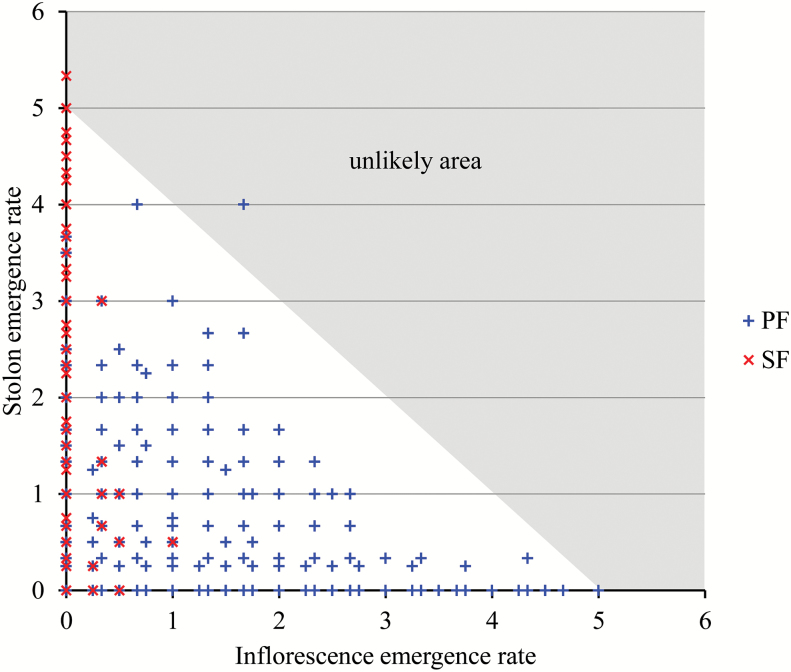
Relation between the inflorescence and the stolon emergence rates distinguishing perpetual flowering (PF) and seasonal flowering (SF) individuals. The area where the combination of inflorescence and stolon emergence rates are very unlikely is indicated.

To investigate the relationship between flowering and vegetative reproduction, we first compared the emergence rates of inflorescences (Fig. 2A) and stolons (Fig. 2B). Production of stolons was much higher and lasted for longer for SF genotypes than for PF genotypes (week 43 for SF genotypes instead of week 35 for most of PF genotypes) (Fig. 2B) and this production was similar among PF and SF genotypes (Supplementary Fig. S2). Considering the first two phases (AIF phase and flowering pause) common to the PF and SF genotypes, we obtained a strongly significant negative Spearman’s rank correlation coefficient between the inflorescence and stolon emergence rates (ρ=−0.57 with *P*<0.01). This simply expresses the successive production of inflorescences (week 19, i.e. beginning of May) and stolons (weeks 21 and 23) (Fig. 2). Considering the perpetual flowering phase (weeks 26, 29, 32, 35, 39, and 43), we obtained a strongly significant negative Spearman’s rank correlation coefficient between the inflorescence and stolon emergence rates (ρ=−0.5 with *P*<0.01) pooling SF and PF genotypes. This coefficient is far lower in absolute value (ρ=−0.2 with *P*<0.01) if only the PF genotypes are considered. In the case of the pooled PF and SF genotypes, the high correlation coefficient (in absolute value) mainly reflects the different habits of the two groups of genotypes, i.e. roughly production of either inflorescences or stolons). The strongly significant negative Spearman’s rank correlation coefficient between the inflorescence and stolon emergence rates (ρ=−0.49 with *P*<0.01) obtained by pooling all the measurement dates and PF and SF genotypes expresses the production of either inflorescences or stolons over time for the first two phases or for the different genotypes during the perpetual flowering phase. It should be interpreted as the expression of a threshold effect characterized by a maximum cumulative number of weekly emerged inflorescences and stolons rather than a monotone relationship between the inflorescence and stolon emergence rates (Fig. 3). These results suggest a strong coordination between these two processes that compete for resources, since one of the two processes is always predominant whatever the period within the growing season or the genotype.

This exploratory analysis focused on the weekly mean numbers of emerged inflorescences and stolons per genotype. This approach did not take into account the potential dependences between the emergences of successive organs (either inflorescence or stolon) for a plant. To decipher the dynamics of the flowering process, we developed a statistical modelling approach for the longitudinal analysis of this process in PF genotypes.

### Segmentation in successive flowering phases highlighted commonalities and differences between the flowering patterns of the PF genotypes

We assumed that the flowering pattern was common for the individuals of a given PF genotype and that this pattern took the form of a succession of well-differentiated stationary flowering phases (e.g. a period of intense flowering followed by a flowering pause). The analysis of these flowering patterns was decomposed into the following two steps.

(i) Segmentation in successive flowering phases, synchronous between the individuals, for each PF genotype. For this analysis of the flowering pattern of each PF genotype, we focused in particular on the selection of the number of flowering phases and on the assessment of the synchronous segmentation assumption.
(ii) Comparison of the segmentations in successive flowering phases of the PF genotypes in order to identify commonalities and differences between the flowering patterns of these genotypes.

For each PF genotype, the number of flowering phases was selected using the ICL criterion. The assumption of a non-ambiguous segmentation in stationary flowering phases of each PF genotype was then assessed. It was in particular assumed that the different individuals of a given PF genotype share the same flowering rate distribution within each phase and the same change-point locations between phases. The flowering series were segmented in three or four flowering phases according to the genotype. For 13 out of the 21 PF genotypes, this corresponded to the number of flowering phases given by the ICL criterion. It should be noted that in our context of short series of length 28, the number of flowering phases given by the ICL criterion should only be considered as indicative. For seven of the remaining genotypes, we selected a more parsimonious model with one less flowering phase (and for CxC_145, with two less flowering phases) with respect to the number of flowering phases given by the ICL criterion. We did not select the number of flowering phases given by the ICL criterion in the following two situations.

(i) Ambiguous segmentation: for CxC_21, CxC_153 and CxC_162 we obtained two markedly different alternative segmentations (e.g. the third change-point between weeks 24 and 25 or between weeks 31 and 32 for CxC_21; see Supplementary Table S2).
(ii) One-week-long flowering phase: we interpreted 1-week flowering phases found for CxC_145, CxC_184, CxC_31, CxC_52 and CxC_150 as a consequence of asynchronisms between individuals of a given genotype. In the segmentation retained for genotype comparison, this 1-week flowering phase is simply merged with one of the two adjacent flowering phases for CxC_184 and CxC_52.

It should be noted that the segmentations used for genotype comparison were always the optimal segmentations in the selected number of flowering phases.

**Fig. 4.**
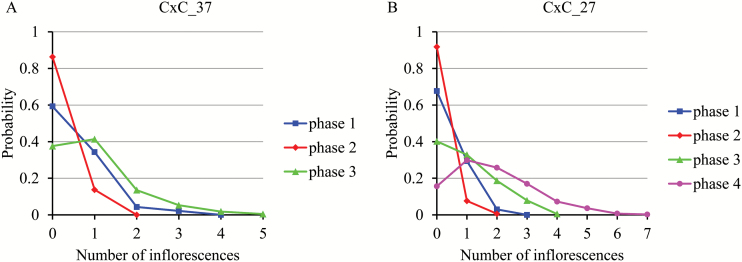
Distributions of the number of the weekly emerged inflorescences for each successive flowering phase: (A) CxC_37; (B) CxC_27.

The distributions of the number of weekly emerged inflorescences for consecutives phases were well differentiated; see a summary in Table 1 and illustrations for CxC_37 (Fig. 4A) and CxC_27 (Fig. 4B). This is consistent with the segmentation assumption. These distributions were right-skewed with a mode at 0 (systematically for phase 2, possibly for the other phases) or 1 (only for phases 1, 3 and 4).

**Table 1.** Segmentation in flowering phases of each PF genotype: flowering phase limits and corresponding mean number of weekly emerged inflorescences (computed without grouping the largest values) The * indicates that the number of flowering phases is the one given by the ICL criterion. If this is not the case, the number of flowering phases given by the ICL criterion is indicated in the next column. The posterior probabilities of the optimal segmentation for the selected number of flowering phases and of the selected multiple change-point model are given.

	Flowering phase^a^											
	1		2		3		4		Posterior probability			
	Limits	Mean	Limits	Mean	Limits	Mean	Limits	Mean	Segment	Model	ICL model	EMFv020 marker
CxC_21	16→18	0.43	19→24	0.06	25→43	0.94			0.45	0.13	4	H
CxC_34	16→19	0.5	20→23	0.07	24→43	0.85			1	0.59*		H
CxC_37	16→19	0.49	20→24	0.14	25→43	0.94			0.99	1*		H
CxC_46^a^	16→17	0.6	18→25	0.15	26→43	0.79			0.57	0.01		H
16→17	0.6	18→25	0.15	26→37	0.62	38→43	1.15	0.41	0.97*			
CxC_145^a^	16→19	0.3	20→24	0.01	25→43	0.88			1	0.01	6	H
16→19	0.3	20→24	0.01	25→35	0.69	36→43	1.13	0.55	0.11			
CxC_152^a^	16→19	0.35	20→24	0.03	25→43	1			1	0.23		A
16→19	0.35	20→24	0.03	25→34	0.89	35→43	1.12	0.38	0.77*			
CxC_161	16→17	0.73	18→23	0.18	24→43	0.96			0.53	0.98*		A
CxC_163	16→18	0.27	19→25	0.04	26→43	0.78			0.8	0.83*		H
CxC_175	16→18	0.46	19→23	0.12	24→43	0.87			0.81	1*		H
CxC_184	16→17	0.51	18→23	0.2	24→43	0.94			0.64	0	4	H
CxC_11	16→19	0.44	20→24	0.02	25→31	0.96	32→43	1.66	0.52	0.99*		A
CxC_27	16→19	0.35	20→24	0.09	25→30	0.96	31→43	1.85	0.52	0.63*		A
CxC_31	16→18	0.41	19→25	0.07	26→36	1.1	37→43	1.65	0.59	0.01	5	A
CxC_52	16→17	0.69	18→25	0.16	26→32	0.91	33→43	1.66	0.5	0.04	5	A
CxC_153	16→19	0.37	20→23	0.08	24→29	0.64	30→43	1.42	0.33	0.03	5	A
CxC_162	16→18	0.33	19→24	0.1	25→29	0.95	30→43	1.67	0.39	0.16	5	A
CxC_174	16→19	0.49	20→25	0.15	26→33	1	34→43	1.62	0.53	1*		A
CxC_196	16→19	0.35	20→24	0.09	25→30	0.85	31→43	1.65	0.46	1*		A
CxC_150^a^	16→17	0.76	18→24	0.3	25→43	1.14			0.98	0	5	?
16→17	0.76	18→24	0.3	25→27	1.69	28→43	1.03	0.98	0.01			
CxC_157	16→19	0.34	20→23	0.03	24→30	0.84	31→43	1.27	0.61	0.96*		H
‘Capitola’	16→17	0.45	18→23	0.05	24→43	1.15			0.76	0.97*		H

^a^ For four genotypes, segmentation in three and four flowering phases was given to ease the comparison of these genotypes with similar three-flowering-phase genotypes.

The assumption of a synchronous segmentation of individuals of a given PF genotype in successive flowering phases is strongly supported by the high posterior probabilities of the selected segmentations. These probabilities were always high: a minimum of 0.33, ≥0.5 for 15 genotypes, ≥0.75 for six genotypes to be related to 351 possible segmentations for three flowering phases and 2925 possible segmentations for four flowering phases.

After building a multiple change-point model for each PF genotype, we compared these models in order to find commonalities and differences between them with the aim of identifying potential groupings of PF genotype flowering patterns. Eighteen out of the 21 genotypes can be classified into two groups according to their number of successive flowering phases (Fig. 4): (i) three flowering phases: CxC_21, CxC_34, CxC_37, CxC_46, CxC_145, CxC_152, CxC_161, CxC_163, CxC_175, CxC_184; and (ii) four flowering phases: CxC_11, CxC_27, CxC_31, CxC_52, CxC_153, CxC_162, CxC_174, CxC_196. For CxC_46, CxC_145 and CxC_152, we give the segmentation in three and four flowering phases in Table 1 to ease the comparison of these genotypes with similar genotypes with three flowering phases. In this case, the segmentation in four flowering phases is a simple refinement of the segmentation in three flowering phases where the PF phase is split into two flowering phases. These three genotypes are far more similar to three-flowering-phase genotypes with a weekly mean number of emerged inflorescences in the late PF phase around 1.1 to be compared with a weekly mean number of emerged inflorescences between 1.42 and 1.85 for the ‘true’ four-flowering-phase genotypes.

The three remaining genotypes CxC_150, CxC_157 and ‘Capitola’ showed flowering patterns intermediate between patterns of the two groups identified previously, the parent ‘Capitola’ being closer to three-flowering-phase genotypes, CxC_157 being in between the two groups, and CxC_150 being atypical.

**Fig. 5.**
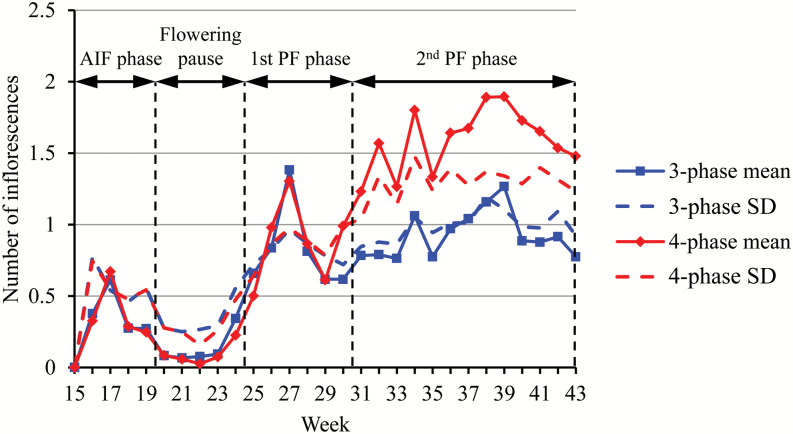
Weekly mean number of emerged inflorescences (and associated standard deviation, SD) for three- and four-flowering-phase individuals. The consensus segmentation in four phases (autumn-initiated flowering (AIF) phase, flowering pause, first and second perpetual flowering phases) is indicated.

The first two flowering phases common between the two groups corresponded respectively to flowering resulting from floral initiation in autumn the previous year (AIF phase) and to a pause of inflorescence emergence (Table 1). The autumn-initiated flowering phase started at week 16 by convention (beginning of observation) while the flowering pause started between weeks 18 and 20 depending on the genotype. The PF phase started between weeks 24 and 26 (Table 1). This PF phase was either stationary (three flowering phases), with a mean of 0.9 weekly emerged inflorescences, or decomposed into two phases of increasing flowering intensity (four flowering phases), with respectively a mean of 0.94 and 1.65 weekly emerged inflorescences. The fourth flowering phase started between weeks 30 and 34 (except for CXC_31 where the fourth flowering phase started week 37). If we compute the weekly mean number of emerged inflorescences for the two groups (Fig. 5), the profiles are very similar up to week 29 and then diverge markedly consistently with the structuring in three or four flowering phases. This confirms the commonality of the first three flowering phases up to week 29. The synchronous fluctuations for the two groups after week 29 (e.g. for weeks 33, 34 and 35) are likely due to changes in the environmental conditions.

### A genomic region different from the *FaPFRU* locus and specific to the PF genotypes controls the occurrence of a second perpetual flowering phase of high intensity

We intended to determine a consensus segmentation of all the plants of the PF genotypes in order to genetically decipher the perpetual flowering process and to identify genetic controls of the different phases of this process using a QTL approach. To this end, we first segmented into three flowering phases the 342 plants of the 10 three-flowering-phase genotypes, and into four flowering phases the 276 plants of the eight four-flowering-phase genotypes. The data thus consisted of a series of dimension 342 or 276 and of length 28. We obtained the three flowering phases 16→19, 20→24 and 25→43 in the first case and the four flowering phases 16→18, 19→24, 25→30 and 31→43 in the second case. In both cases, the only segmentation ambiguity concerned the first change point, which can be either between weeks 18 and 19 (posterior segmentation probability of 0.24 for the three flowering phases and of 0.5 for the four flowering phases) or between weeks 19 and 20 (posterior segmentation probability of 0.64 for the three flowering phases and of 0.45 for the four flowering phases). We thus chose as a consensus segmentation for QTL detection the four flowering phases: 16→19, 20→24, 25→30 and 31→43. This consensus segmentation is indeed highly consistent with the segmentations of the PF genotypes shown in Table 1 (Fig. 5).

We used this consensus segmentation to identify genomic regions responsible for the changes in flowering emergence rates at well-defined time instants. A QTL approach was conducted using the linkage maps previously obtained (Gaston *et al.*, 2013) and analyses were performed separately for the number of inflorescences or stolons newly emerged during the four flowering phases. In addition, emergence of stolons during the identified flowering phases was also considered taking account of the link between flowering and runnering. Depending on whether the analyses were conducted on both PF and SF genotypes or on PF genotypes only, different QTLs associated with the control of the dynamics of perpetual flowering were identified (Table 2). When QTL analysis was conducted on both PF and SF genotypes, the major locus, *FaPFRU* (Gaston *et al.*, 2013), localized on the female (F) linkage group (LG) 4b, LG4b_F, was identified whatever the PF phase (25→30, 31→43). This QTL, responsible for the switch between SF and PF (Gaston *et al.*, 2013), had a positive effect on perpetual flowering and a negative effect on runnering (Table 2).

**Table 2.** Map positions and genetic effect of significant QTL detected for flowering segmentation traits for the female (_F) parent ‘Capitola’ and for the male (_M) parent ‘CF1116’ The QTL identification was based on composite interval mapping analysis with LOD > LOD threshold (α=0.01). Bold shows the QTL localized on the LG3c and linked to the fourth phase of intense flowering.

Linkage group	QTL name^*^a^*^	QTL previously identified	Marker^*^b^*^	LOD^*^c^*^	*r*^2 *^d^*^	Effect^*^e^*^
**SF and PF 28 genotypes**						
Total period of observations (all weeks)						
LG1a_M	Ru_Week_all_SF&PF		tgag395	6.2	31	+
** LG3c_F**	**Flo_Week_all_SF&PF**		**EMFv020_146**	**6.2**	**10**	**−**
LG4b_F	Flo_Week_all_SF&PF	*FaPFRU*	gatt284	16.1	75	−
LG4b_F	Ru_Week_all_SF&PF	*FaPFRU*	gatt284	20.8	97	−
LG4d_M	Flo_Week_all_SF&PF		tgta115	5.8	61	−
Third segment of flowering (25→30)						
LG4b_F	Flo_Week25_30_SF&PF	*FaPFRU*	gatt284	15	61	+
LG4b_F	Ru_Week25_30_SF&PF	*FaPFRU*	gatt284	16.1	87	−
LG7c_M	Flo_Week25_30_SF&PF		ccta282c	6.1	86	−
Fourth segment of flowering (31→43)						
LG4b_F	Flo_Week31_43_SF&PF	*FaPFRU*	gatt284	9.7	31	+
LG4b_F	Ru_Week31_43_SF&PF	*FaPFRU*	gatt284	20	96	−
LG7c_M	Ru_Week31_43_SF&PF		ccta282c	11	87	+
**Exclusively PF 21 genotypes**						
Total period of observations (all weeks)						
** LG3c_F**	**Flo_Week_all_SF&PF**		**EMFv020_146**	**8.5**	**70**	**−**
LG5a_M	Ru_Week_all_SF&PF		UDF003_128	5.3	49	+
Third segment of flowering (25→30)						
LG4d_F	Flo_Week25_30_PF		tcac304	5.9	49	−
LG5a_F	Flo_Week25_30_PF		tcaa355	5.1	41	+
Fourth segment of flowering (31→43)						
** LG3c_F**	**Flo_Week31_43_PF**		**EMFv020_146**	**9.2**	**67**	**−**

^*a*^ Flo: flowering; Ru: runnering; SF: seasonal flowering; PF: perpetual flowering.

^*b*^ The left marker associated with QTL is indicated.

^*c*^ LOD is the log-likelihood at that position.

^*d*^ *r*^2^ is the percentage of phenotypic variation explained by the QTL.

^*e*^ Effect on a trait mean value of the presence of one allele at a marker by comparison with the presence of the second allele. + and − indicate the direction of the additive effect. A positive effect means a higher value for ‘Capitola’ allele on the female map or a higher value for ‘CF1116’ on the male map.

Surprisingly, this major QTL was not identified when analyses were conducted exclusively on PF genotypes (Table 2), while a strong QTL associated with a late PF intense phase (31→43) was identified on LG3c_F (named hereafter LG3c_locus). Together, these results suggested different genetic controls for PF, where one is associated with the switch between SF and PF and another with the occurrence of a second perpetual flowering phase of high intensity. Besides the two loci, *FaPFRU* and LG3c_locus, other QTLs were identified on the female linkage groups LG4d_F and LG5c_F for the first PF phase (25→30) in PF analyses and on the male linkage group LG7c_M. The two localized on LG7c_M showed opposite effects, positive on flowering in the period (25→30) and negative on runnering in the period (31→43).

### The absence of the allele EMFv020_146 localized near the maximum of the LG3c_F QTL is associated with a late intense PF phase in six consecutive years of growing

An interesting output from the longitudinal data analysis was the temporal effect of the marker EMFv020_146 localized near the maximum of the QTL on LG3c_F. The absence of this marker was linked to a positive effect on the occurrence of a second PF phase of high intensity, while its presence did not modify the dynamics of PF leading to a unique stationary PF phase (Table 2). The effect of this allele was further supported by the absence of this marker in the eight four-flowering-phase genotypes, while the three-flowering-phase genotypes except CxC_152 and CxC_161 were characterized by the presence of allele (H).

These results were strengthened by the analyses on the PF individuals of the entire segregating population issued from the cross between ‘Capitola’ and ‘CF1116’ using previous data obtained from 2004 to 2009. We cumulated the number of emerged inflorescences from August to October (2004, 2005, 2007, 2008) or November (2006, 2009) and compared the number of inflorescences between PF individuals having or not having the EMFv020_146 marker. These six years of observation showed contrasted climatic conditions with warmer summers such as in 2006 or colder summers such as in 2007 and 2008 (Supplementary Fig. S3). The results showed a significant negative effect of the presence of the allele (Student’s *t*-test *P*<0.05). This effect led to a decrease from 1.8–4.9 inflorescences according to the year with respect to individuals without the allele (Supplementary Table S3) in the late PF phase. These results confirm the presence of a locus localized on LG3c linked to the occurrence of a second PF phase of high intensity.

### The numbers of inflorescences, stolons and crowns produced during a growing season are consistent with a strong coordination between flowering and runnering processes

In order to both validate the results of the longitudinal data analysis and investigate the developmental role of crowns, we explored the counts of the different organs (inflorescences, stolons and crowns) at the end of the growing seasons and their relationships. The mean numbers of inflorescences were ranked according to the PF genotype grouping deduced from the longitudinal data analysis: between 15.2 and 21.8 for the three-flowering-phase genotypes (mean of 19.2), between 25.4 and 31.6 for the four-flowering-phase genotypes (mean 27.9) and intermediate (between 23.9 and 25.2) for the three remaining PF genotypes (Table 3). The number of inflorescences produced during the growing season was not significantly different (*P*-value of 0.12 for the ANOVA) between three-flowering-phase genotypes if the two genotypes of lowest mean number of inflorescences (CxC_46 and CxC_163) were excluded. The number of inflorescences was not significantly different (*P*-value of 0.15 for the ANOVA) between four-flowering-phase genotypes if the genotype of highest mean number of inflorescences (CxC_27) was excluded. The number of inflorescences was always significantly different between each three-flowering-phase genotype and each four-flowering-phase genotype. This ranking of the mean numbers of inflorescences produced during the growing season is indeed highly consistent with the grouping deduced from the longitudinal data analysis using multiple change-point models. Concerning the mean number of stolons, there was a strong overlap between the three- and the four-flowering-phase genotypes (see Supplementary Fig. S4 for the relation between the number of inflorescences and the number of stolons for three- and four-flowering-phase genotypes).

**Table 3.** Final number of inflorescences, stolons and crowns and cumulative number of inflorescences, stolons and crowns (mean and standard deviation (SD) for each of these four count variables) for each genotype and for the pooled samples (in bold) corresponding to three- and four-flowering-phase perpetual flowering (PF) genotypes, for perpetual flowering genotypes and for seasonal flowering (SF) genotypes

Genotype	No. of inflorescences		No. of stolons		No. of crowns		Cumulative number	
	Mean	SD	Mean	SD	Mean	SD	Mean	SD
CxC_21	19.5	4.9	9.8	4	4	1.5	33.3	8.3
CxC_34	19.2	5.4	4.7	2	4.1	1.7	28	6.4
CxC_37	20.5	4.9	3.8	1.8	3.7	1.5	28	6.4
CxC_46	16.7	7.5	5.6	2	3.9	2	26.2	9.2
CxC_145	17.9	5.2	10.8	4	5.2	1.9	33.9	7.2
CxC_152	20.5	4.8	5	1.7	3.9	1.9	29.4	6.1
CxC_161	21.8	6.2	5.4	2.1	2.9	1.1	30.1	7.7
CxC_163	15.2	6.6	9.3	3.2	4.4	2.1	28.9	7.4
CxC_175	19.5	5.6	5.1	1.8	3	1.7	27.6	7.5
CxC_184	20.9	6.5	3.6	1.3	3.3	1.5	27.8	6.6
**Three-phase PF**	**19.2**	**6.1**	**6.3**	**3.5**	**3.8**	**1.8**	**29.3**	**7.6**
CxC_11	28.6	6.6	8.7	2.9	8	2.4	45.3	8.3
CxC_27	31.6	4.7	6.7	2.6	7.3	2.7	45.6	7.1
CxC_31	25.4	6.5	6.7	2.3	5.5	2.1	37.6	8.3
CxC_52	27.3	7.2	11.5	4.2	8.2	2.9	47	11
CxC_153	25.5	7.2	8.8	3.2	7	3.6	41.3	10.7
CxC_162	29.8	9.8	7	2.4	4.9	2.6	41.7	12.3
CxC_174	27	7.7	5.3	2.4	6.1	2.3	38.4	10.5
CxC_196	28.4	7.1	6.6	2.6	6.5	1.9	41.5	8.5
**Four-phase PF**	**27.9**	**7.4**	**7.7**	**3.4**	**6.7**	**2.8**	**42.3**	**10.1**
CxC_150	25.2	7.8	2.6	1.4	4.2	1.9	32	8.4
CxC_157	23.8	4.6	5.6	1.6	4.4	1.8	33.8	5.8
‘Capitola’	24.3	7.4	5.4	1.7	3.9	1.3	33.6	7.8
**PF genotypes**	**23.3**	**7.8**	**6.6**	**3.5**	**5**	**2.6**	**34.9**	**10.6**
‘CF1116’	1.6	0.7	23.7	6	4	1.8	29.3	6.2
CxC_22	0.9	0.2	28.8	8.2	4.1	1.7	33.8	9.2
CxC_36	1	0	38.7	8.3	5.2	1.4	44.9	8.6
CxC_39	0.8	0.5	30	6.9	4.7	2.2	35.5	8.1
CxC_47	0.9	0.3	32.8	6.3	4.9	1.3	38.6	6.9
CxC_108	0.9	0.3	31.1	10.4	3.8	2.2	35.8	11.3
CxC_135	1.7	1	27.4	5.3	5.8	2	34.9	6.1
**SF genotypes**	**1.1**	**0.6**	**30.4**	**8.6**	**4.6**	**1.9**	**36.1**	**9.3**

For PF genotypes, the number of crowns was positively correlated with the number of stolons (significant linear correlation coefficient of 0.34) and with the number of inflorescences (significant linear correlation coefficient of 0.47) (Supplementary Fig. S5). In the latter case, we suspect that the emergence of supplementary crowns at a given time may explain the burst in inflorescence emergence for four-flowering-phase genotypes. For SF genotypes, the number of crowns was positively correlated with the number of stolons (significant linear correlation coefficient of 0.32). The mean cumulative numbers of inflorescences, stolons and crowns, which can be assimilated to the mean numbers of meristems activated during the growing season, were ranked according to the PF genotype grouping deduced from the longitudinal data analysis: between 26.2 and 33.9 for the three-flowering-phase genotypes (mean of 29.3), and between 37.6 and 47 for the four-flowering-phase genotypes (mean of 42.9). The three intermediate genotypes (between 32 and 33.8) were very close to the two three-flowering-phase genotypes of highest mean values (33.3 for CxC_21 and 33.9 for CxC_145).

It should be noted that the cumulative numbers of inflorescences, stolons and crowns were not significantly different between PF and SF genotypes (*P*-value of 0.1 for the ANOVA). The number of meristems activated during the growing season was therefore similar in the two main groups of genotypes despite their contrasting modes of development.

## Discussion

We present evidence that, in the field, PF is a highly structured non-stationary process composed of successive phases synchronous between individuals of a given genotype. By studying its dynamics using a statistical modelling approach together with a QTL approach, we identified genetic keys that control PF. In addition to the locus *FaPFRU* controlling the switch between SF and PF (Gaston *et al.* 2013; Perrotte *et al.*, 2016), we identified a locus on LG3c associated with an abrupt change in floral initiation during PF leading to a split of PF into two phases, the second one being more intense.

### Relevance of longitudinal data analysis for investigating the dynamics of developmental processes

Perpetual flowering is usually investigated on the basis of one-off measures rather than longitudinal measures. These one-off measures may be the number of weeks for the duration of flowering (*Arabis alpina*: Albani *et al.*, 2012), the presence of flowers at different dates of the growing season (rose and woody strawberry: Iwata *et al.*, 2012; cultivated strawberry: Weebadde *et al.*, 2008) or a count of flowers at the end of a flowering phase (strawberry: Honjo *et al.*, 2011). The same remark is valid for other developmental processes such as root growth or branching (Liu *et al.*, 2011; van Norman *et al.*, 2014).

Because of its non-stationary character, PF cannot be summarized by a global indicator. It was thus critical in this study to design an efficient approach for the longitudinal analysis of PF data. The analysis of complex plant longitudinal data is a difficult issue and improper analyses often lead to erroneous biological conclusions. This can be illustrated by the flowering profiles shown in Fig. 1 and Supplementary Fig. S1, which only convey part of the information related to the flowering pattern. The weekly means computed from a set of flowering series (Fig. 1 and Supplementary Fig. S1) are not appropriate to identify successions of flowering phases since the potential dependencies between the emergence of successive inflorescences for a plant are not taken into account in such an approach. This approach is referred to as a cross-sectional study in statistics, the alternative approach where the individual series is directly considered being referred to as a longitudinal study (Diggle *et al.*, 2002). In particular, since the flowering phases were defined by categorical distributions with some dispersions (Fig. 4) and the phase changes were not synchronous between PF genotypes (and also to a lesser extent between plants for a given genotype), the changes in mean in Fig. 1 and Supplementary Fig. S1 reflect both dispersion of the flowering variable within phase and asynchronisms between flowering series. It is thus essential to apply proper longitudinal data analysis methods to identify flowering patterns. Various segmentation models have been previously applied to identify growth or developmental phases at different scales in plants (see e.g. Dambreville *et al.*, 2015; Lièvre *et al.*, 2016; Guédon *et al.*, 2007). By identifying successive synchronous phases in flowering and by showing that the split into two perpetual flowering phases can be genetically explained, this study illustrates the relevance of statistical models for better characterizing dynamic developmental traits using longitudinal analyses of plant phenotyping data.

### Segmentation models highlighted different genetic controls of the perpetual flowering process

In this study, we showed that a statistical modelling approach based on multiple change-point models allowed us to identify a succession of flowering phases. Using data specific to each flowering phase, we identified two noteworthy QTLs linked to a succession of flowering phases. The first one is the already known major locus that controls the PF trait, *FaPFRU* (Gaston *et al.*, 2013) and was identified on the basis of one-off measures (Castro *et al.*, 2015). The second one, LG3c_locus identified in this study, controls the occurrence of a late PF phase of high intensity. At this locus, the absence of the allele EMFv020_146 is associated with a fourth flowering phase in PF genotypes, during which almost 1.6 inflorescences were produced per week while less than one inflorescence was produced during the previous phase in almost all PF genotypes.

In addition to genetic control, environmental conditions can modify the expression of the PF trait (Battey *et al.*, 1998; Heide *et al.*, 2013), and a segregating population can show more PF genotypes than expected in a warm environment (Maltoni *et al.*, 1996). However, stability over years of these two QTLs related to PF traits in strawberry was confirmed by analysing the entire segregating population over 6 years in Gaston *et al.* (2013) for *FaPFRU* and here for the LG3c_locus. Stability over genetic background could be further studied by analysing genetic resources (Horvath *et al.*, 2011).

**Fig. 6.**
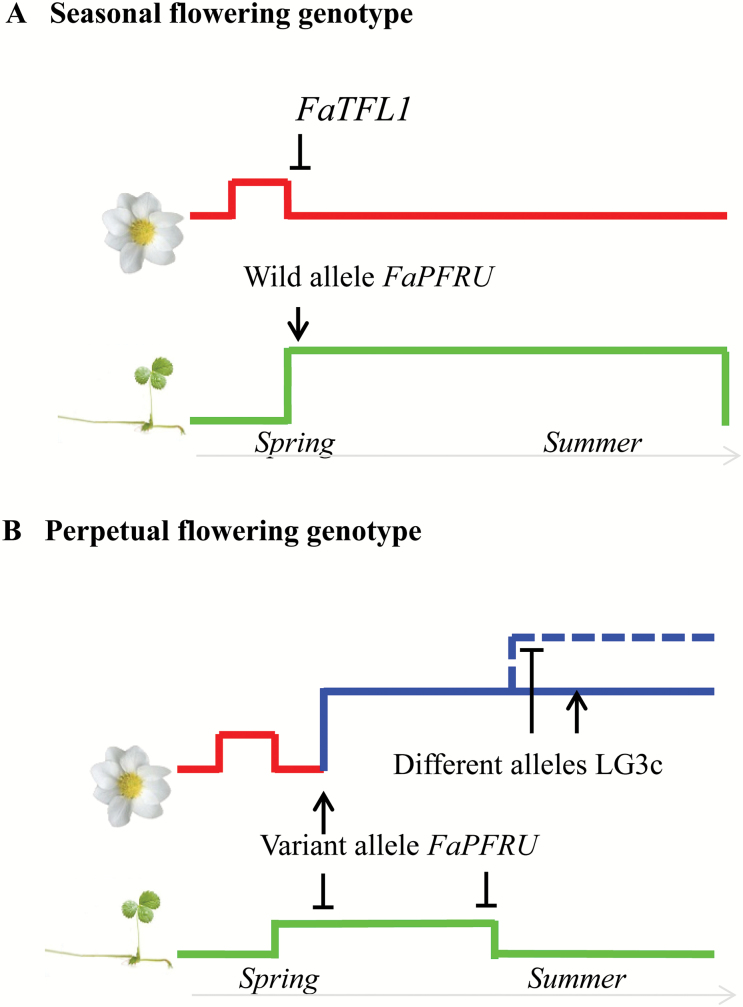
Model of genetic control of perpetual flowering (PF) in strawberry. (A) In seasonal flowering (SF) genotypes, the ‘wild’ allele *FaPFRU* leads to a typical SF pattern with only the autumn-initiated flowering phase in spring and several synchronous fluctuations of emergence of primary stolons mainly between June and September. In these SF genotypes, *FaTFL1* acts as a floral repressor in long days (Koskela *et al.*, 2016). (B) In PF genotypes, the ‘variant’ allele *FaPFRU* leads to a typical PF pattern. This allele acts as a floral activator, which should overcome the floral repressor *FaTFL1* in long days. According to the allelic status of the LG3c_locus, the PF phase is stationary or is splitted into two PF phases, the second one being more intense. The emergence of primary stolons in PF genotypes is low and occurs mainly between June and August.

Based on the two genetic controls linked to PF, we are able to propose a model for flowering in octoploid strawberry (Fig. 6). In wild type plants (SF phenotype), in the absence of the ‘allelic variant’ of the *FaPFRU* gene (presence of ‘wild type alleles’ of the *FaPFRU* gene), the floral repressor *FaTFL1* should repress the transition to reproductive development under long days (Koskela *et al.*, 2016), which promotes stolon production (Fig. 6A). To the contrary, in PF plants (Fig. 6B), one single copy of an ‘allelic variant’ of the *FaPFRU* gene is sufficient to promote continuous flowering, which limits the subsequent stolon production. This ‘allelic variant’ acts as a floral activator, which should overcome the floral repressor *FaTFL1* under long days. Then, PF genotypes displayed a single stationary PF phase (three-flowering-phase genotypes) or two PF phases, the second one being more intense (four-flowering-phase genotypes), according to the allele status of the LG3c_locus, which is linked to the marker EMFv020.

The LG3c_locus in addition to *FaPFRU* offers a new basis for breeding programmes by selecting or not the PF habit (*FaPFRU*) with or without a late intense PF phase. Producers should consider whether they will contribute or not to reach new niche markets by planting genotypes having a late production phase of high intensity.

### When did flower initiation take place?

The emergence of inflorescences from the crown results from the transition of the shoot apical meristem (SAM), which becomes floral after floral induction and initiation (Jahn and Dana, 1970). In our study, we can clearly separate flowering phases according to their period of floral initiation: floral initiation of the first flowering phase (AIF phase) occurred in the previous autumn, while floral initiation of the perpetual flowering phases (the two last phases) occurred the same year. To evaluate the time when the floral initiation began for the PF phase, we assumed that the time lag between floral initiation of terminal meristem and visual emergence of inflorescences was between 8 and 10 weeks in strawberry [e.g. about 10 weeks for the SF ‘Frida’ variety (Sonsteby and Heide, 2008), and 9 weeks for the SF ‘Elsanta’ variety (Battey *et al.*, 1998)]. Based on this lag, we can hypothesize that the floral initiation leading to the PF phase started toward the end of the AIF phase, 2–3 weeks after planting (i.e. at the middle of April) (Fig. 1).

### A possible role for the LG3c_locus linked to the late intensive perpetual flowering in shoot branching

In strawberry branching is sympodial, with floral initiation occurring terminally. Extension axes can develop from the uppermost axillary buds below the terminal inflorescence (Battey *et al.*, 1998) or more occasionally in the basal part of the primary crown (Sugiyama *et al.*, 2004), giving birth to new crowns with terminal flowering. Since a higher number of crowns was observed in four-flowering-phase genotypes (Table 3 and Supplementary Fig. S5), we can hypothesize that the LG3c_locus controls, at least partially, the branching of the plant leading to a higher number of branches (crowns) and therefore to a higher number of shoot apical meristems becoming floral. This branching control should take place after planting since almost all plants displayed a single crown at planting.

Branching is under the control of numerous pathways (e.g. gibberellins) that converge to probable common integrators like BRC1 (review in Rameau *et al.* 2015). In strawberry, gibberellins strongly influence the shoot branching by playing a role in the photoperiodic control of axillary bud differentiation (Hytönen *et al.* 2009) and present an interesting pool of candidate genes to explain the influence of the LG3c_locus. Before identifying the gene underlying the LG3c_locus, we needed first to narrow down this locus. For this purpose, we developed a strategy based on selective mapping associated to the QTL approach (Perrotte *et al.*, 2016). We applied this strategy to the *FaPFRU* locus and identified candidate genes for this locus. Among them a *FaFT2* gene appears an excellent candidate since FT is known to be the florigen for numerous species (Corbesier *et al.*, 2007).

### The plant results from a competitive investment among vegetative and sexual reproduction

Inflorescence and stolon developments takes place in different pools of meristems, respectively terminal and basal (Costes *et al.*, 2014). Despite the distant location of these pools, balance in resource allocation between sexual and vegetative reproduction was well supported by negative correlations. This competitive investment among vegetative and sexual reproduction (e.g. Obeso, 2002) resulted in a threshold on the maximum cumulative number of inflorescences, stolons and crowns that emerged during a given period. This threshold is suggested by an approximately similar number of meristems in SF and PF genotypes, which become floral or vegetative according to the status of *FaPFRU* locus and which can be increased according to the status of the LG3c_locus. Hormonal (Guttridge and Thompson, 1964) and environmental factors such as temperature and photoperiod (Heide *et al.*, 2013), known to alter the balance between vegetative and floral development in strawberry (Durner *et al.*, 1984; Bradford *et al.*, 2010), may further contribute to the changes in inflorescence and stolon emergence rates. Another competitive investment could occur between the AIF and PF phases, separated by a phase in which flowering almost ceases, such as observed in tomato between successive trusses (Bertin *et al.*, 2003).

## Conclusions

Many wild plant species are characterized by more than one reproductive strategy, including sexual breeding strategies (e.g. outcrossing or self-fertilizing) and asexual strategies (e.g. vegetative propagation). Under cultivation, however, only one of these strategies is usually exploited as a propagation method for a given species. Both sexual and vegetative reproduction are concerned for perennial crop species such as strawberry since vegetative reproduction by stolons is used in nurseries to multiply varieties of interest and sexual reproduction is a key event for fruit production. Our results provide evidence that a longitudinal data analysis, here based on segmentation models, enables the deciphering of complex dynamic developmental traits such as flowering in PF genotypes. A direct extension would be to consider multivariate longitudinal data combining vegetative development, flowering and runnering variables with the same time indexing. This would allow the identification of global developmental phases relying on the different developmental processes in competition within plants.

## Supplementary data

Supplementary data are available at *JXB* online.

Figure S1. Weekly mean number of emerged inflorescences for each genotype.

Figure S2. Pointwise mean number of weekly emerged inflorescences and number of weekly emerged stolons for each genotype.

Figure S3. Climatic data from 2004 to 2009 and in 2011 and from May to November.

Figure S4. Relation between the number of inflorescences and the number of stolons distinguishing three- and four-flowering-phase genotypes.

Figure S5. Relation between the number of inflorescences and the number of crowns distinguishing three- and four-flowering-phase genotypes.

Table S1. Phenotypic data measured from 2002 to 2007 for the 28 selected genotypes.

Table S2. Segmentations and associated posterior probabilities corresponding to the number of flowering phases given by the ICL criterion when these segmentations were not retained for genotype comparison.

Table S3. Comparison between individuals showing (H) or not (A) the EMFv020_146 marker linked to the inflorescence emergence intensity during the late perpetual flowering phase.

Supplementary Data
