# Supplementary material for: Identification of successive flowering phases highlights a new genetic control of the flowering pattern in strawberry
Source: J Exp Bot. 2016 Sep 24;67(19):5643–55. doi: 10.1093/jxb/erw326 (PMC5066487; doi:10.1093/jxb/erw326)
Supplement: Supplementary Data [file supp_67_19_5643__index.html]

Identification of successive flowering phases highlights a new genetic control of the flowering pattern in strawberry — Identification of successive flowering phases highlights a new genetic control of the flowering pattern in strawberry — Supplementary Data 

# Identification of successive flowering phases highlights a new genetic control of the flowering pattern in strawberry

## Supplementary Data

Data files

- Supplementary\_figures\_S1\_S5.pdf - Supplementary Data
- Supplementary\_tables\_S1\_S3.pdf - Supplementary Data
