# Supplementary material for: Identification of successive flowering phases highlights a new genetic control of the flowering pattern in strawberry
Source: J Exp Bot. 2016 Sep 24;67(19):5643–55. doi: 10.1093/jxb/erw326 (PMC5066487; doi:10.1093/jxb/erw326)

Journal of Experimental Botany Supplementary Data - Figures

Article title: Identification of successive flowering phases highlights a new genetic control of the flowering pattern in strawberry

Authors: Justine Perrotte, Yann Guédon, Amèlia Gaston, Béatrice Denoyes

The following Supporting Information is available for this article:

Fig. S1 Weekly mean number of emerged inflorescences for each genotype

Fig. S2 Pointwise mean number of weekly emerged inflorescences and number of weekly emerged stolons for each genotype.

Fig. S3. Climatic data from 2004 to 2009 and in 2011 and from May to November.

Fig. S4 Relation between the number of inflorescences and the number of stolons distinguishing three- and four-flowering-phase genotypes.

Fig. S5 Relation between the number of inflorescences and the number of crowns distinguishing three- and four-flowering-phase genotypes.

Figure S1. Weekly mean number of emerged inflorescences for each genotype. The parents Capitola and CF1116 are respectively in blue with squares and red with diamonds.

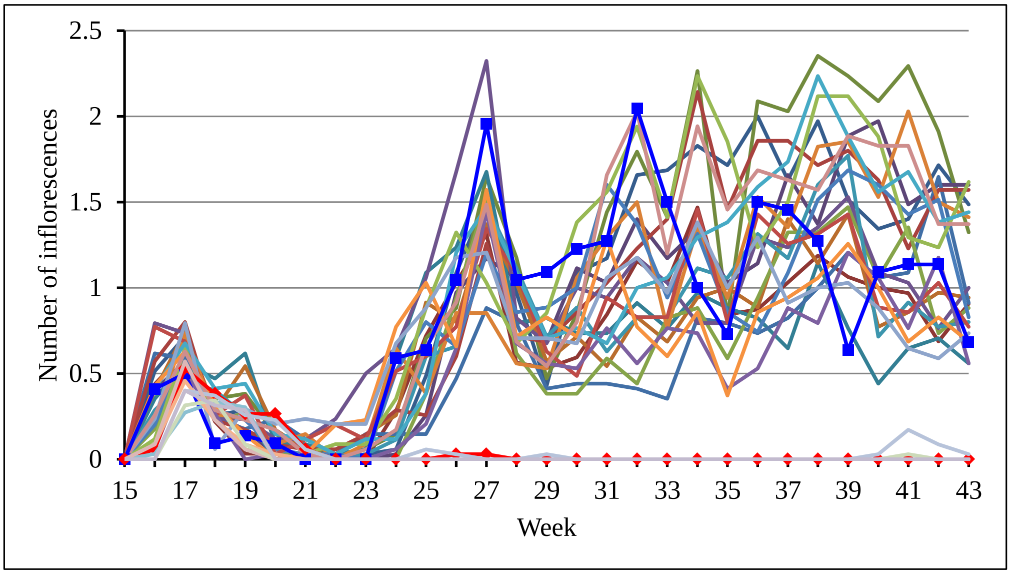

Figure S2. Pointwise mean (A) number of weekly emerged inflorescences and (B) number of weekly emerged stolons for each genotype. The common indexing of these mean and standard deviation series is the sparser stolon time indexing. The parents Capitola and CF1116 are respectively in blue with squares and red with diamonds.

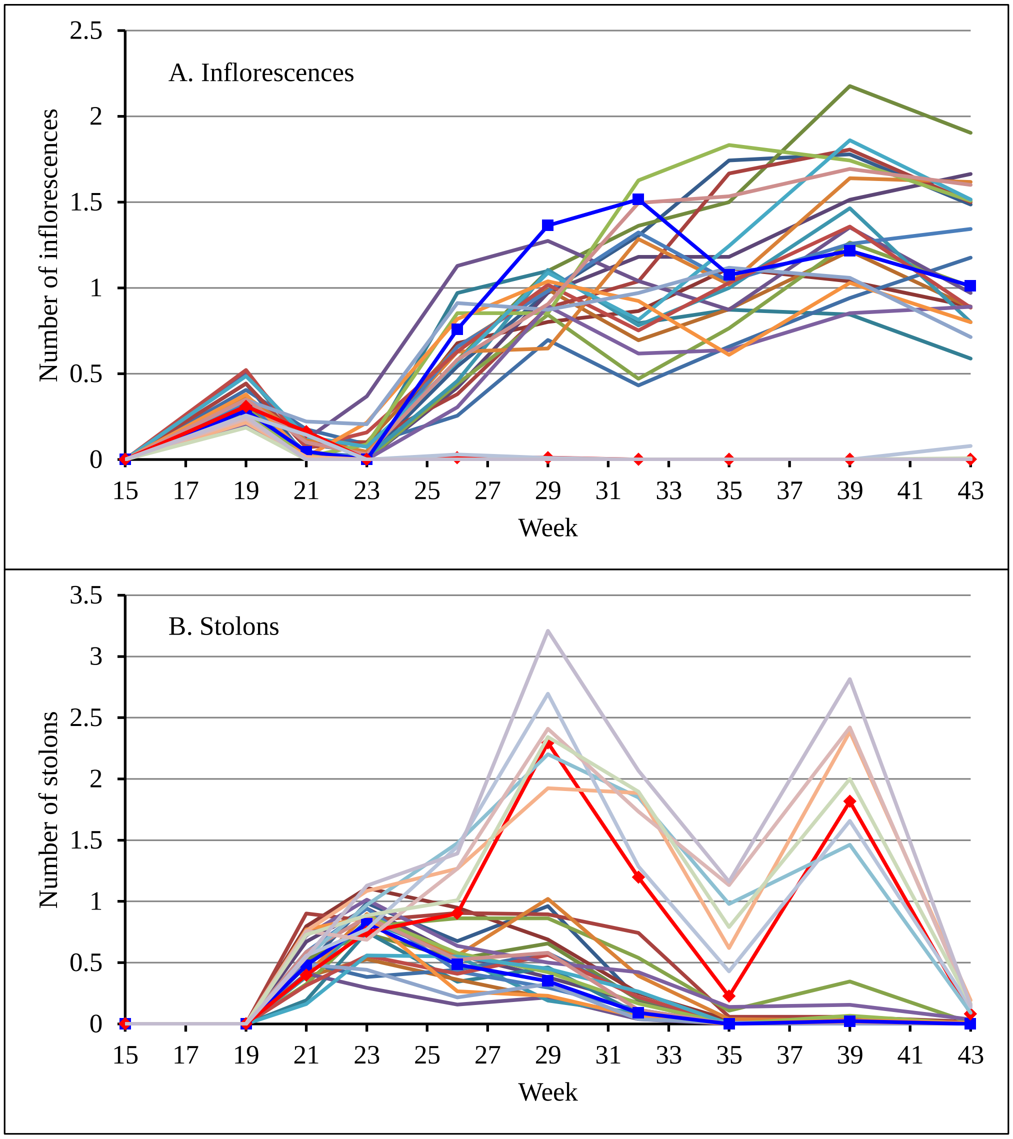

Figure S3. Climatic data from 2004 to 2009 and in 2011 and from May to November. Arrows indicate the date when inflorescences were cut. Late period of inflorescences was recorded as number of inflorescences from September to October or November according the year.

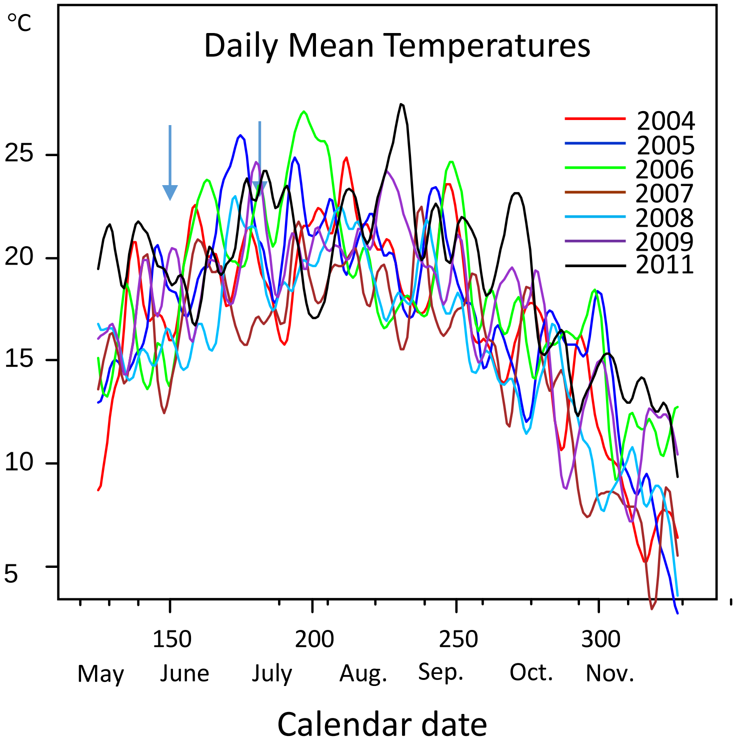

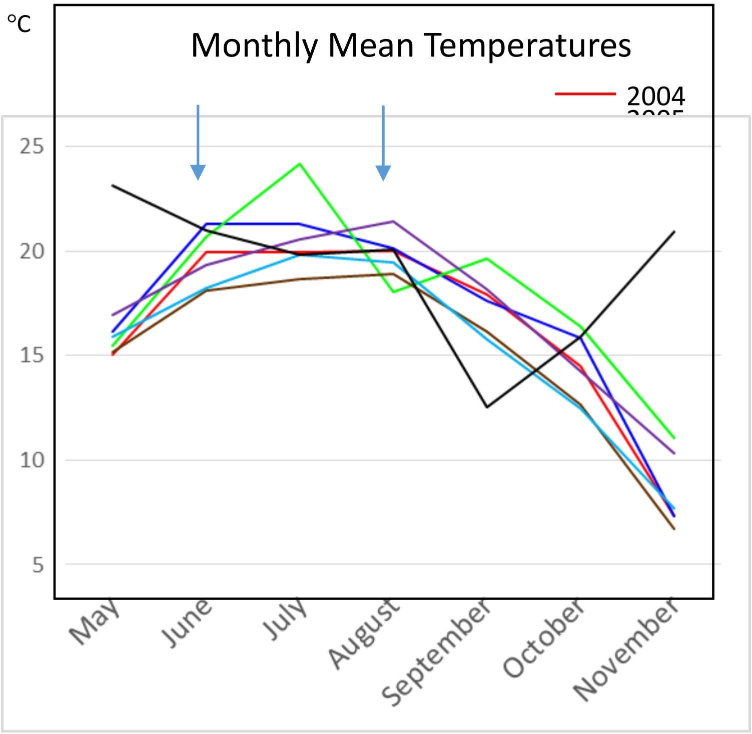

Figure S4. Relation between the number of inflorescences and the number of stolons distinguishing three- and four-flowering-phase genotypes

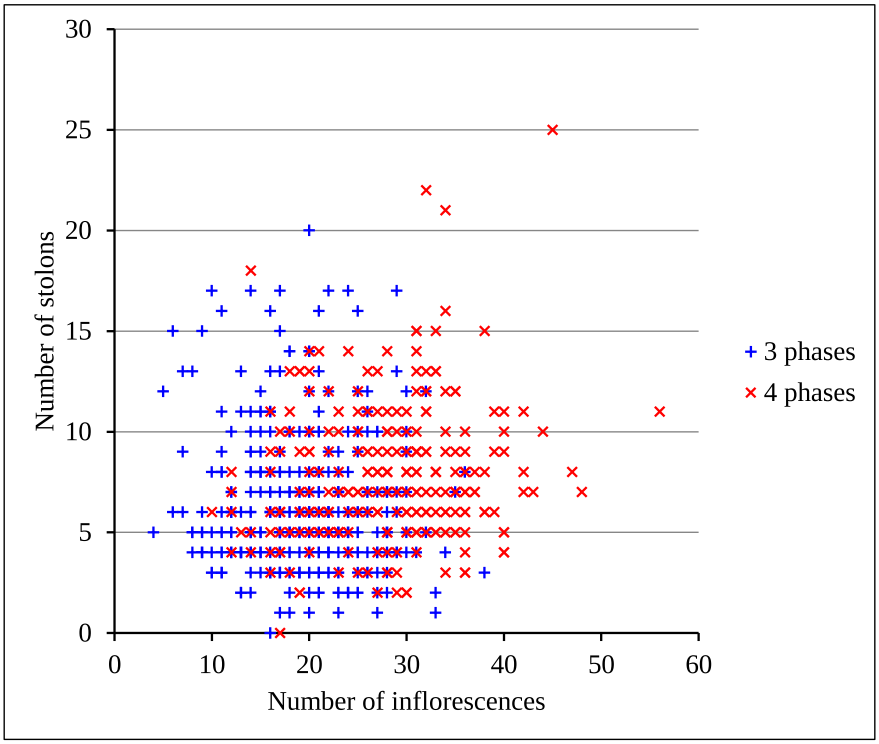

Figure S5. Relation between the number of inflorescences and the number of crowns distinguishing three- and four-flowering-phase genotypes

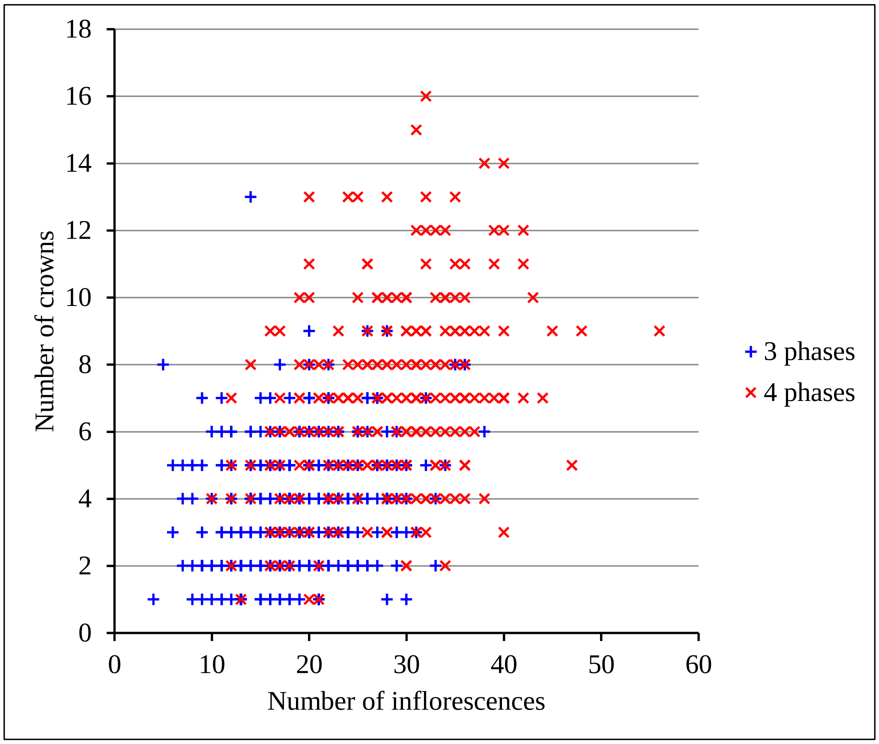

Supplement: Supplementary Data [file supp_erw326_Supplementary_figures_S1_S5.pdf]
