# Supplementary material for: Identification of successive flowering phases highlights a new genetic control of the flowering pattern in strawberry
Source: J Exp Bot. 2016 Sep 24;67(19):5643–55. doi: 10.1093/jxb/erw326 (PMC5066487; doi:10.1093/jxb/erw326)
Supplement: Supplementary Data [file supp_erw326_Supplementary_tables_S1_S3.pdf]

Journal of Experimental Botany Supplementary Data - Tables

Article title: Identification of successive flowering phases highlights a new genetic control of the flowering pattern in strawberry

Authors: Justine Perrotte, Yann Guédon, Amèlia Gaston, Béatrice Denoyes

Table S1 Phenotypic data measured from 2002 to 2007 for the 28 selected genotypes.

Table S2 Segmentations and associated posterior probabilities corresponding to the number of flowering phases given by the ICL criterion when these segmentations were not retained for genotype comparison.

Table S3 Comparison between individuals showing (H) or not (A) the EMFv020_146 marker linked to the inflorescence emergence intensity during the late perpetual flowering phase.

Table S1. Phenotypic data measured from 2002 to 2007 for the 28 selected genotypes

Genotype	Number of inflorescences measured at end-July or beginning-August						Number of stolons summer	
	2002	2003	2003b	2004	2005	2007	2002	2003
Perpetual flowering gentoypes								
CxC_11	8.0	11.0	11.3	12.0	5.7	15.0	3.0	0.0
CxC_21	14.0	10.0	7.8	8.5	13.0	11.7	7.0	1.0
CxC_27	28.0	16.0	23.3	15.0	12.0	11.7	6.0	3.0
CxC_31	11.0	9.0	12.0	14.3	6.0	12.7	6.0	2.0
CxC_34	8.0	5.0	15.0	9.5	9.0	12.0	4.0	0.0
CxC_37	7.0	13.0	10.3	12.3	13.0	16.0	6.0	3.0
CxC_46	3.0	6.0	14.0	7.3	8.0	13.5	7.0	0.0
CxC_52	12.0	-	18.3	7.8	16.0	8.0	2.0	0.0
CxC_145	9.0	6.0	12.5	5.3	15.7	-	7.0	0.0
CxC_150	5.0	2.0	11.3	16.0	6.0	-	4.0	0.0
CxC_152	11.0	13.0	12.0	11.3	9.7	16.5	5.0	0.0
CxC_153	6.0	10.0	15.0	11.7	17.7	11.0	8.0	0.0
CxC_157	7.0	11.0	14.7	11.8	7.0	2.0	6.0	9.0
CxC_161	8.0	4.0	9.3	7.5	12.0	2.7	6.0	0.0
CxC_162	7.0	6.0	15.3	12.0	20.5	9.3	7.0	0.0
CxC_163	8.0	9.0	10.3	12.8	15.5	6.7	7.0	0.0
CxC_174	14.0	6.0	6.3	15.0	7.0	6.0	7.0	1.0
CxC_175	21.0	24.0	18.3	10.5	4.5	16.0	5.0	0.0
CxC_184	22.0	7.0	9.5	14.7	7.3	4.5	7.0	0.0
CxC_196	17.0	15.0	20.0	14.8	14.0	12.5	7.0	1.0
Capitola	21.3	-	21.8	12.5	3.8	6.1	-	0.4
Seasonal flowering gentoypes								
CxC_22	0.0	1.0	0.0	0.3	0.0	0.0	8.0	14.0
CxC_36	0.0	0.0	0.8	0.3	0.0	1.7	7.0	13.0
CxC_39	0.0	0.0	1.3	0.8	0.0	0.0	8.0	5.0
CxC_47	0.0	4.0	0.3	1.0	0.0	3.0	7.0	8.0
CxC_108	0.0	5.0	0.3	0.3	0.0	0.0	8.0	20.0
CxC_135	0.0	1.0	2.3	1.1	0.3	4.0	6.0	1.0
CF1116	1.2	-	1.3	2.0	0.3	1.0	-	5.0

Table S2. Segmentations and associated posterior probabilities corresponding to the number of flowering phases given by the ICL criterion when these segmentations were not retained for genotype comparison. Ambiguities regarding segmentation and one-year-length flowering phases are in boldface.

	Flowering phase						Posterior
Genotype	1	2	3	4	5	6	probability
CxC_21	16 → 18	19 → 23	**24 → 24**	**25 → 43**			0.39
	16 → 18	19 → 23	**24 → 31**	**32 → 43**			0.16
CxC_145	16 → 19	20 → 24	**25 → 25**	26 → 27	28 → 35	36 → 43	0.68
CxC_184	**16 → 16**	**17 → 17**	18 → 23	24 → 43			0.57
CxC_31	16 → 19	20 → 24	**25 → 25**	26 → 36	37 → 43		0.91
CxC_52	**16 → 16**	**17 → 17**	18 → 25	26 → 32	33 → 43		0.51
CxC_153	**16 → 16**	**17 → 17**	**18 → 23**	24 → 29	**30 → 43**		0.33
	**16 → 19**	**20 → 23**	24 → 29	**30 → 35**	**36 → 43**		0.09
CxC_162	**16 → 16**	**17 → 17**	**18 → 24**	25 → 29	30 → 43		0.61
	**16 → 18**	**19 → 23**	**24 → 24**	25 → 29	30 → 43		0.16
CxC_150	16 → 17	18 → 22	23 → 25	26 → 27	28 → 43		0.93

Table S3. Comparison between individuals showing (H) or not (A) the EMFv020_146 marker linked to the inflorescence emergence intensity during the late perpetual flowering phase.

	2004[a]	2005[a]	2006[a]	2007[a]	2008[a]	2009[a]
Individuals A	16.50	11.45	14.04	17.95	20.59	11.99
Individuals H	12.62	9.63	11.35	13.61	15.72	9.32
Effect of presence of marker	-3.88	-4.82	-2.69	-4.34	-4.87	-2.67
Student's *t*-test	0.005	0.043	0.013	0.005	0.018	0.035

[a]Late perpetual flowering trait was evaluating by cumulating the number of emerged inflorescences from August to October (2004, 2005, 2007, 2008) or to November (2006, 2009).
